# Psychosocial Workloads and Resilience of Heads of Municipal Public Health Authorities in Germany During the COVID-19 Pandemic: Perceptions of Operational Organization, Communication, and Measures

**DOI:** 10.3390/ijerph21111421

**Published:** 2024-10-26

**Authors:** Veit Kinne, Sabine Trommer, Dragisa Mitic, Sandra Ehrenberg, Annette Jurke, Nora-Lynn Schwerdtner, Astrid van der Wall, Nicoletta Wischnewski, Frank Kipp

**Affiliations:** 1 Institute for Infectious Diseases and Infection Control, Jena University Hospital, 07747 Jena, Germany; sabine.trommer@med.uni-jena.de (S.T.); dragisa.mitic@med.uni-jena.de (D.M.); frank.kipp@med.uni-jena.de (F.K.); 2 Corporate Development, Medical University of Lausitz—Carl Thiem, 03048 Cottbus, Germany; s.ehrenberg@mul-ct.de; 3 NRW Centre for Health, Infectious Disease Epidemiology, 44802 Bochum, Germany; annette.jurke@lzg.nrw.de; 4 Saxon State Ministry for Social Affairs and Cohesion, 01097 Dresden, Germany; nora-lynn.schwerdtner@sms.sachsen.de; 5 Center for Rare Diseases, Jena University Hospital, 07747 Jena, Germany; astrid.vanderwall@med.uni-jena.de; 6 District Office Charlottenburg-Wilmersdorf of Berlin, Public Health Authority, 10713 Berlin, Germany; cw500000@charlottenburg-wilmersdorf.de

**Keywords:** municipal public health authorities, psychosocial workload, resilience resources, organizational resilience, COVID-19 pandemic, crisis management, outreach teams

## Abstract

Healthcare professionals are particularly vulnerable to mental health issues during epidemics, as evidenced by the COVID-19 crisis. German public health authorities, crucial for disease prevention, faced significant strain from chronic understaffing and resource limitations exacerbated by the pandemic. The study was designed as a cross-sectional, observational online survey. This study conducted an online needs assessment survey among heads of municipal public health authorities in Thuringia, Saxony, North Rhine-Westphalia, Bavaria, and Berlin between June and November 2023. Of the 191 contacted authorities, 74 responses (38.7%) were analyzed, focusing on professional demands, recognition, stress resilience, general life satisfaction, operational organization, and communication during the pandemic. Validated scales such as ERI, RS-13, L-1, and the COVID-19 add-on module of the COPSOQ were utilized. Statistical tests included descriptive statistics, correlation coefficients, Chi-Square tests, linear regression, T-tests, and ANOVA with a significance level set at *p* < 0.05. Respondents were mainly from North Rhine-Westphalia (43.3%) and Bavaria (24.3%), predominantly female (54.1%), and had a mean age of 52.7 years. The majority were medical specialists (71.9%). The RS-13 mean score was 72.66 (*SD* = 12.42), with 58.9% demonstrating high stress resilience. Public health degree holders showed the highest resilience. The ER-ratio indicated high effort versus reward for 96.7% of heads. Larger districts showed lower ER-ratios, suggesting resilient organizational structures. The study highlights high psychosocial workload and resilience among German public health authority heads during COVID-19, suggesting the need for optimized crisis management and scalable staffing for future pandemics and crises.

## 1. Introduction

Findings from epidemics show that healthcare professionals are at risk of developing short- and long-term mental health problems [1]. The World Health Organization (WHO) has warned of the potential negative impact of the COVID-19 crisis on the mental well-being of health and social care professionals [1]. German public health authorities play a key role in the prevention of communicable diseases [2]. The essential importance of municipal public health authorities in regional pandemic and crisis management was known long before the pandemic, but despite numerous references, recommendations, and resolutions [3,4], it was not reflected in the necessary personnel or material resources of these authorities. In the decades before the pandemic, the development of the German public health service was characterized by staff cuts and general savings, not only in terms of personnel and material resources, but also in the context of the professional qualifications of employees in this sector. The COVID-19 pandemic hit the German public health service that had been weakened for decades and was not adequately and specifically prepared with regard to pandemic and crisis management. This drastically changed the working and ultimately also the living conditions of employees of German public health authorities within a very short space of time. The heads of these authorities were particularly challenged here, as they had to respond to this unforeseen reaction in the shortest possible time in terms of organization, personnel, and materials, and were therefore under particular strain. The COVID-19 pandemic took variable shapes and forms, in terms of cases and deaths, in different regions and countries of the world [5]. The COVID-19 pandemic showed that every country remains vulnerable to public health emergencies [5]. Gerlinger et al. (2021) demonstrated that public health services in Sweden, France, and Austria differ significantly [6]. The study shows that France has a highly centralised health system, while Sweden’s system is strongly regional and municipal. These structural differences have led to different approaches to pandemic management [6].

Based on a systematic literature review by Brooks et al. (2018), the psychological impact of SARS, in the context of the SARS outbreak in 2002, on healthcare professionals was related to job role, training, high-risk work environments, quarantine, perceived risk, social support, social rejection/isolation, and the impact on personal or professional life [7]. Symptoms of mental health problems typically include depression, anxiety, stress, and additional cognitive and social problems; these can affect the function of the workplace [1]. The mental health and resilience of heads of German public health authorities could have been supported during the COVID-19 pandemic through workplace measures (e.g., training, changing routines or measures) to support basic daily professional needs [1]. The most common description of the term “resilience” is the human ability to withstand stressful life circumstances and thus represents a positive counter-concept to vulnerability [8]. Therefore, it is about the process, the ability, or the result of successfully coping with stressful life circumstances [8]. There are also other approaches of defining resilience. For example, another definition is stress resistance, which assumes that people have developed an immunity to stress [9].

Psychosocial stress is most commonly experienced in the workplace as a result of challenges in a demanding environment that are difficult to cope with and therefore cause intense negative feelings and alarming physiological reactions due to the risk of failure [10]. Work intensification, job insecurity, poor quality of work, and pay inequality are among the main causes of workplace stress [11]. Siegrist’s model of the professional gratification crisis was used in this study to depict the work load of the heads of the municipal public health authorities. It results from the experienced disparity between the effort put in and the reward received for it (pay, recognition, job security, and promotion opportunities) [12]. Gratification crises are triggered, for example, when opportunities for promotion are blocked and the pay is perceived as unfair or the workload as too high. This leads to feelings of loss of control and powerlessness, which in turn poses a health risk [13,14]. According to the model, gratification crises always arise when one’s own commitment/effort is not compensated by a corresponding reward, such as job security, remuneration, opportunities to help shape the work, or similar [13]. Changing working conditions due to demographic change have an impact on the general quality of life of doctors, respectively, heads of municipal public health authorities, as well as on the working atmosphere, job satisfaction, and workload [13]. The shortage of material and human resources, combined with the demand to do more in less time, has an increasing impact on the working atmosphere, health, and quality of life of doctors [15].

The present study is intended to contribute to the recording and understanding of the workload of heads of municipal public health authorities from different German federal states during the COVID-19 pandemic, which challenged the municipal public health authorities to an unprecedented extent. In addition, the intrinsic resilience and general life satisfaction of these heads were surveyed. The communication and operational organization of these heads were also reviewed. The following hypotheses can be derived from this study:

1. The surveyed heads of municipal public health authorities from different German federal states perceive varying levels of professional demands (effort) and professional recognition (reward) across regions.
2. There is a significant tendency among the heads of municipal public health authorities to overcommitment at work.
3. The stress resilience of the surveyed heads of municipal public health authorities varies significantly across the German federal states.
4. The resilience of the surveyed heads moderates the effect of occupational stress and influences the outcomes based on their district or administrative area.
5. The psychosocial workload, resilience, and operational organization and communication of the surveyed heads differ significantly based on socio-demographic factors such as gender, age, and qualifications.
6. The general life satisfaction of the respondents varies significantly between the German federal states.
7. The surveyed heads reported positive influences during the COVID-19 pandemic on internal exchange, crisis support, emotional support, and working atmosphere within the municipal public health authorities.
8. The surveyed heads differ in their ratings of internal crisis communication, the sense of security provided by measures, and their individual crisis awareness in their respective public health authorities.
9. The surveyed heads were involved in the decisions of the municipal task forces and assumed that outreach teams can provide support for future crises.

In addition, the intrinsic resilience and general life satisfaction of the heads were assessed. The heads’ communication and operational organization were also examined.

This study provides valuable insights into the psychosocial challenges and resilience of public health authority heads in Germany during the COVID-19 pandemic. By identifying key stressors, organizational demands, and the relationship between effort, reward, and resilience, the findings can inform targeted interventions for improving mental health and operational efficiency in public health settings. Furthermore, the study underscores the importance of optimizing crisis management strategies and addressing chronic staffing shortages, which are critical for enhancing the preparedness and response capacity of public health authorities in future pandemics or similar crises. These results offer a foundation for evidence-based policy recommendations aimed at sustaining the well-being and effectiveness of public health professionals, who are essential to safeguarding public health.

## 2. Methods

### 2.1. Participants and Data Collection

From June 2023 to November 2023, a needs assessment based on an online questionnaire was conducted among the heads of municipal public health authorities in Thuringia, Saxony, North Rhine-Westphalia, Bavaria, and Berlin. The study employs a cross-sectional, observational online survey targeting the heads of municipal public health authorities across five German states. Its objective is to evaluate professional demands, resilience, and work stress (psychosocial workload) levels during the COVID-19 pandemic. Data was collected using validated scales, including ERI-Short, RS-13, L-1, and the COPSOQ-COVID-19 module, and analyzed through both descriptive and inferential statistical methods. A total of 191 municipal public health authorities were contacted, and a large proportion of the questionnaires were incomplete. The average response rate after the data set cleansing was 38.7%. The questionnaire consisted of 76 items. Only the validated scales were used in this study. Heads who did not provide basic demographic and social information or did not fully complete the measurement instruments were excluded. Only fully completed cases were considered for evaluating the measurement instruments used. Therefore, the sample size (*n*) may vary.

LimeSurvey was used as the online survey tool. The aim of the survey was to evaluate processes and to identify typical problems related to pandemic and crisis management in German municipal public health authorities during the COVID-19 pandemic. In addition, the heads were asked to develop possible solution strategies for better crisis management (e.g., evaluating the usefulness of outreach teams). As a first step, the workload and the associated stress, stress resilience, general life satisfaction, operational organization, and communication, as well as operational measures, were retrospectively examined from the perspective of the heads during the COVID-19 pandemic. An additional file shows this in more detail (see Additional File S1).

To estimate the sample size, the following assumptions were made: significance level α = 0.05, power 80%, mean of the ER-ratio (psychosocial workload) of German employees based on ERI-Short according to Li et al. (2019); ER-ratio *M* = 0.97, *SD* = 0.46 [16]. To detect a difference between the groups of heads from the different federal states of 5.0 on ER-ratio (corresponds to an effect size of *f* = 0.25 [17,18], a total of 200 heads and 40 heads per group are required. For the heads of municipal public health authorities, the required number of 200 people was not reached after the data set was cleaned up. After data cleansing, 74 questionnaires were used for this study. The required number of 200 heads of municipal public health authorities was not reached, despite participation from five different federal states and the use of reminder letters.

### 2.2. Questionnaire

#### 2.2.1. Socio-Demographic Data

The questionnaire provided socio-demographic data on the heads, such as age, gender, and qualifications, as well as the number of inhabitants of the cities/districts/counties to be supported by the relevant authority. 

#### 2.2.2. Effort-Reward Imbalance (ERI) at Work

In order to evaluate the psychosocial workload, the short form of the Effort-Reward Imbalance (ERI) scale, adapted to the work environment (ERI at work), developed by Siegrist [19], was used. The short form of the ERI at work scale consists of a total of 16 items with the scales effort (ERI1 to ERI3), reward (ERI4 to ERI10), and overcommitment (OC1 to OC6) [19]. The reward scale at work is divided into the three subscales, in particular esteem (ERI4, ERI8), job security (ERI6, ERI7), and job promotion (ERI5, ERI9, ERI10) [12,19]. The job overcommitment scale reflects excessive work-related commitment [16]. The German version of the ERI questionnaire was validated and tested in the general population [20]. The ER-ratio at work was calculated by dividing the total (sum) score of the effort scale (numerator, E) by the sum score of the reward scale (denominator, R); the denominator was multiplied by a correction factor (c) to compensate for the unequal number of items in the effort and reward scales [12]. ER-ratio values greater than 1 indicate that effort exceeds reward, and values less than 1 indicate that reward exceeds effort [12]. Siegrist et al. (2019) reported reliable psychometric properties for the ERI scale [21]. The German version of the ERI questionnaire 13 was utilized for this study.

#### 2.2.3. Stress Resilience (RS-13)

The resilience scale RS-13 by Leppert et al. (2008) was applied to measure the construct of resilience [22]. This scale consists of 13 individual items that are to be answered on a seven-point Likert scale ranging from level 1 (strongly disagree) to level 7 (strongly agree) [22]. The evaluation is done by adding the point values [23]. The following scheme was used to interpret the characteristics of the resilience classes: 13 to 66 points as low, 67 to 72 points as moderate, and 73 to 91 points as high resilience [23]. The psychometric quality of the resilience scale has been confirmed in studies [24,25]. The RS-13 is recognized as a reliable, valid, and economical tool for identifying individuals in challenging situations who are at increased risk of impairments, particularly in mental health, due to low resilience [26]. Various short versions have been developed for German-speaking regions, with the 13-item version (RS-13) being the most commonly used in research [27]. The German version of the RS-13 was utilized for this study.

#### 2.2.4. General Life Satisfaction (L-1)

An 11-point rating scale (general life satisfaction L-1) is available to answer the question on general life satisfaction. Life satisfaction is understood as part of the concept of subjective well-being [28]. The response categories of the L-1 range from “not at all satisfied” (0) to “completely satisfied” (10) [28]. The L-1 was validated and standardized [28]. An average value between 0 and 10 is formed for the evaluation. Reference values for the L-1 are available [28]. Beierlein et al. (2015) developed reference values for the L-1 based on a population-representative random sample, presented as group means and standard deviations [28]. The scale demonstrates a retest reliability of 0.67, and there is evidence supporting both content validity and construct validity [28]. The German version of the L-1 was utilized for this study.

#### 2.2.5. COVID-19 Add-On Module of the COPSOQ

The “COVID-19 add-on module” of the Copenhagen Psychosocial Questionnaire (COPSOQ) is an instrument specially designed to assess the psychosocial impact of the COVID-19 pandemic and is suitable for both scientific questions and risk assessment in companies [29,30]. The add-on module supplements the regular COPSOQ questionnaire with questions that address the specific challenges and stresses associated with the COVID-19 pandemic [29]. These questions typically address issues such as fear of infection, social isolation, changes in the work environment, and the perceptions of employer support [29]. In this study, the “organization and communication” scale was used. It distinguishes between the dimensions “internal exchange”, “support in crisis”, “emotional support”, and “working atmosphere” [29]. A further scale, “operational measures and overall assessment”, was included. In this scale, questions about “internal crisis communication”, “feeling of security through measures”, and “individual crisis awareness” are answered with the aspects “workplace as a risk” and “crisis-related stress” [29]. The two scales consist of nine individual items, which are to be answered on a Likert scale from level 0 (very low) to level 100 (very high) [29]. The COVID-19 add-on module of the COPSOQ has undergone validation. It was incorporated into existing COPSOQ surveys, and data from over 30,000 respondents were analyzed [31,32]. This validation enables the assessment of psychological burdens and the effectiveness of operational measures during the pandemic. The German version of the COVID-19 add-on module of the COPSOQ was utilized for this study.

### 2.3. Statistical Analysis

In this study, the socio-demographic characteristics of the heads were analysed first. The results of the ERI at work, the RS-13, the L-1 and the COVID-19 add-on module scale of the COPSOQ were examined using mean values and standard deviations. In a further step, correlation coefficients were used to analyse the relationships between the socio-demographic characteristics of the heads and the scales used. All bivariate relationships between workplace factors and outcome factors were assessed in correlation analyses. The Chi-Square test was used for correlations and associations. Simple linear regression anal-yses were used to test relationships between the measures used. The *T*-test and the one-factorial analysis of variance (ANOVA) were used to compare mean values. All statistical analyses were performed using IBM SPSS (Statistical Pack for the Social Sciences) version 29.0 (SPSS Inc., Chicago, IL, USA). Statistical significance was set at *p* < 0.05.

## 3. Results

### 3.1. Socio-Demographic Data

Most of the heads came from North Rhine-Westphalia with 43.3%, followed by 24.3% from Bavaria, 16.2% from Thuringia, and 8.1% each from Saxony and Berlin (Table 1). A total of 54.1% of the heads were female, 44.6% male, and 1.3% diverse/miscellaneous (Table 1). The average age of the heads at the time of the survey was 52.7 years (*M* = 52.77, *SD* = 9.30). 55.4% of the heads were aged 55 and over, followed by the 45–54 age group with 23.0% (Table 1).

In terms of medical qualifications, 71.9% of the heads reported having a degree as a medical specialist for public health care, 67.2% had other medical specialist qualifications, and 17.2% had a degree in public health. The average number of medical qualifications reported was 1.5. This was a multiple response set. The Chi-Square test was performed between gender and the multiple response set of medical qualifications of the heads. The correlation was statistically non-significant between gender (excluding diverse) and medical qualifications (χ^2^(3) = 5.16, *p* = 0.160). 

**Figure 1:**
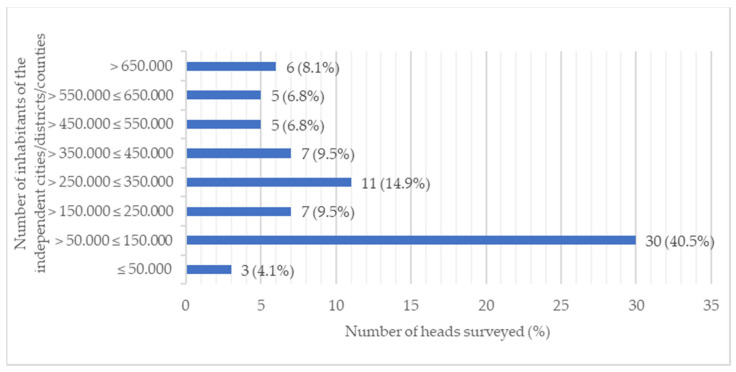
Structural characteristics of the heads (*n* = 74).

Most heads were employed in cities, urban districts, and counties with a population of >50,000 ≤ 150,000 (40.5%), followed by >250,000 ≤ 350,000 (14.9%), >350,000 ≤ 450,000 (9.5%) and >150,000 ≤ 250,000 (9.5%) (Figure 1). When comparing the structural characteristics of the heads (Figure 1) at the federal state level, 91.7% of the heads from Thuringia oversaw cities, city districts, and counties with populations between 50,000 and 150,000, followed by 77.8% of the heads from Bavaria. In contrast, 83.3% of the department heads from Berlin managed city districts with populations between 350,000 and 450,000. Heads from North Rhine-Westphalia primarily oversaw cities, city districts, and counties with populations between 250,000 and 350,000, accounting for 28.1%.

### 3.2. Stress Resilience According to RS-13

To determine the internal consistency, Cronbach’s alpha was calculated for 13 items with the RS-13. The internal consistency was excellent, with Cronbach’s alpha = 0.90 for the RS-13 [33]. To further characterize the RS-13, mean values, standard deviations, and discriminatory power were calculated at item level (Table 2).

The RS-13 mean (total score) was *M* = 72.66 (*SD* = 12.42), i.e., the heads had moderate to high stress resilience. An RS-13 mean score of *M* = 73.14 (*SD* = 8.48) was achieved by female heads (*n* = 28) and male heads (*n* = 27), (*M* = 74.37 (*SD* = 10.55). There was no statistically significant difference between the resilience (RS-13 mean score) and the two sexes (*t*(53) = −0.476, *p* = 0.636). 

Looking at the individual resilience classes, 58.9% of the heads showed a high level of stress resilience, 25.0% a low level, and 16.1% a moderate level. The lowest calculated point value of the RS-13 was 13 and the highest was 91, which, according to the scheme of Leppert et al. (2008), represents a low and high characteristic for resilience, respectively [22].

**Figure 2:**
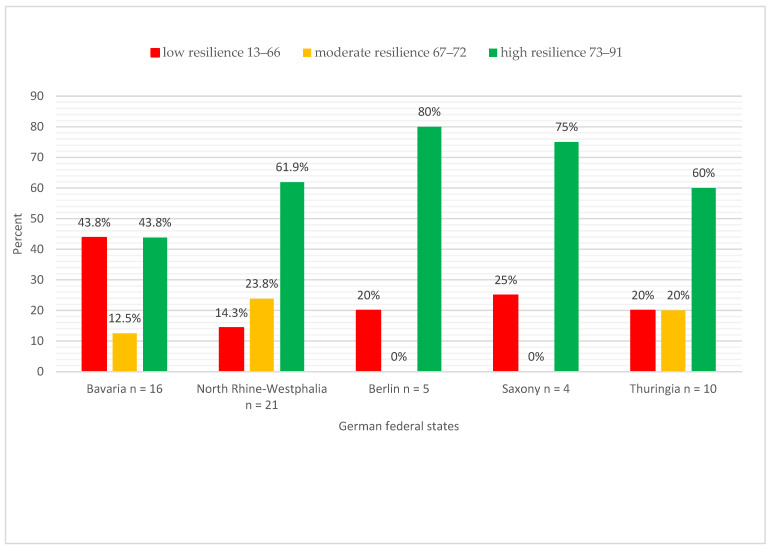
Distribution of heads according to resilience classes at federal state level (*n* = 56).

The heads from Berlin (80%), Saxony (75%), North Rhine-Westphalia (61.9%), and Thuringia (60.0%) showed a high level of resilience (Figure 2). In contrast, heads from Bavaria showed a heterogeneous picture with low (43.8%) and high (43.8%) resilience (Figure 2). There is a significant positive correlation between the RS-13 mean value and the number of inhabitants to be supervised in the cities/urban districts/counties (Figure 1), Spearman’s ρ = 0.341, *p* = 0.010. According to Cohen (1988), this is a moderate effect [34]. This means that a higher RS-13 mean value, and thus a greater expression of the resilience characteristic, occurs for a head with a larger district/county to supervise. When the resilience classes are considered at the level of qualifications, the highest resilience is found among heads with a degree in public health (*M* = 73.49, *SD* = 8.99), followed by heads with other medical specialist qualifications (*M* = 72.82, *SD* = 13.44) and heads with a medical specialist qualification for public health care (*M* = 70.74, *SD* = 13.81). The correlations were statistically non-significant between the variables RS-13 and medical specialist qualification for public health care (*r* (54) = −0.201; *p* = 0.137), other medical specialist qualifications (*r* (54) = 0.16; *p* = 0.904), and degree in public health (*r* (54) = 0.028; *p* = 0.839). There was also no significant difference between the age groups (25 years to 55 years and older) and the RS-13 mean (*F*(3, 51) = 0.110; *p* = 0.954). 

### 3.3. General Life Satisfaction According to L-1

According to the reference values of the L-1, a higher mean value of general life satisfaction could be determined for the surveyed heads (*M* = 7.70, *SD* = 1.86) than in the population-representative random sample of Beierlein et al. (2015) with a high level of education (*M* = 7.47, *SD* = 1.92) [28]. In contrast, the female heads (*M* = 7.57, *SD* = 1.95) had a slightly lower mean value than the random sample representative of the population (*M* = 7.59, *SD* = 1.93). The male heads (*M* = 7.74, *SD* = 1.78) had a higher mean value than the random sample representative of the population (*M* = 7.34, *SD* = 1.91). However, there was no significant difference between the L-1 and both sexes (*U* = 390.00, Z = 0.208, *p* = 0.835). If the L-1 mean value of the heads is considered at federal state level, the heads of all four federal states had higher mean values, except for the Thuringian heads (*M* = 7.20, *SD* = 2.86), compared to the population-representative random sample. The L-1 correlates significantly positively with the RS-13 (Spearman’s, ρ = 0.504, *p* < 0.001). According to Cohen (1988), this is a large effect. Furthermore, the L-1 and the overcommitment scale (Table 3) correlated moderately negatively with each other (Spearman’s, ρ = −0.316, *p* = 0.018), and the L-1 correlated moderately positively with the esteem subscale (Table 3) (Spearman’s, ρ = 0.361, *p* = 0.006). The correlation was statistically non-significant between the level of education or qualification of the heads and life satisfaction (*r* = −0.095, *p* = 0.486; *r* = 0.127, *p* = 0.352; *r* = −0.023, *p* = 0.868).

### 3.4. Psychosocial Workload According to ERI at Work

Cronbach’s alpha was calculated to determine the internal consistency of the ERI at work scale. The internal consistency was acceptable, with Cronbach’s alpha = 0.682 [33]. The Cronbach’s alpha was good for the effort subscale (α = 0.805), acceptable for reward (α = 0.719), and also acceptable for overcommitment (α = 0.730) [33].

The sum score for effort at work was *M* = 11.45 (*SD* = 1.26). Accordingly, the psychosocial workload was high for the heads, and they perceived increased stress experiences. The sum score for reward at work was *M* = 18.34 (*SD* = 3.96). Accordingly, the heads perceived moderate professional rewards (range 7–28). Reward at work has a positive effect on the heads’ resilience (b = 0.979; *p* = 0.017) and has a share in the explanation of variance of *R*^2^ = 0.102, *F*(1, 54) = 6.12; *p* = 0.017. For overcommitment at work, the mean value was *M* = 19.23 (*SD* = 3.62) (Table 3). This indicates a higher propensity to overcommitment (range 6–24). Within the reward subscales, the total score for esteem was *M* = 5.32 (*SD* = 1.69), for job security *M* = 5.74 (*SD* = 1.34), and for job promotion *M* = 7.27 (*SD* = 2.15) (Table 3). In the gender comparison of the heads, men made slightly more effort than women (Table 3). Male heads had a significantly higher reward than female heads (Table 3). Women had a slightly higher mean value than men for overcommitment (Table 3). The mean value of the ER-ratio at work was *M* = 1.54 (*SD* = 0.50) (Table 3). This indicates that the heads with an ER-ratio > 1 put in more professional effort, on average, for each professional reward. A total of 96.7% of the heads (60) had an ER-ratio > 1. This ER-ratio > 1 can be interpreted as a critical value in terms of psychosocial stress intensity, as the effort exceeds the expected reward. Female heads (*M* = 1.61, *SD* = 0.63) showed the highest ER-ratio at work compared to their male colleagues (*M* = 1.47, *SD* = 0.31) (Table 3). There were no significant gender differences for the effort (*t*(59) = −0.747, *p* = 0.458), reward (*t*(59) = −1.36, *p* = 0.177), overcommitment (*t*(59) = −0.379, *p* = 0.706) scales or for the ER-ratio at work (*t*(59) = −1.10, *p* = 0.272). There was no difference between the age groups (25 years to 55 years and older), and the ER-ratio *F*(3, 56) = 2.10, *p* = 0.889.

The highest ER-ratio of heads at the German federal state level was found for Thuringia (*M* = 1.72, *SD* = 0.81), followed by Bavaria (*M* = 1.56, *SD* = 0.44), North Rhine-Westphalia (*M* = 1.50, *SD* = 0.44), Berlin (*M* = 1.42, *SD* = 0.20), and Saxony (*M* = 1.39, *SD* = 0.24). The calculated ER-ratio did not differ significantly with respect to the level of the German federal states *F*(4, 57) = 0.537, *p* = 0.709. There was a negative correlation between the ER-ratio of the heads and the size of the urban districts and rural districts supervised, *r* = −0.273, *p* = 0.032. The negative correlation indicates that larger urban and rural districts are associated with lower ER-ratio. There was a moderate correlation between the ER-ratio and the overcommitment of the heads, *r* = 0.485, *p* < 0.001 (see Additional File S2). 

When the ER-ratio is considered at the level of head’s qualification, the heads had similar ER-ratio values (ER-ratio > 1). Heads with a medical specialist qualification for public health care had an ER-ratio of *M* = 1.55 (*SD* = 0.39). Heads with another medial specialist qualification reported an ER-ratio of *M* = 1.54 (*SD* = 0.53), and heads with a degree in public health had an ER-ratio of *M* = 1.51 (*SD* = 0.14).

Head’s perceived psychosocial workload (ER-ratio at work) did not correlate significantly with their resilience (RS-13) (Pearsons, *r* = −0.151, *p* = 0.268). 

### 3.5. Operational Organization and Communication as Well as Operational Measures and Overall Assessment

The scales company organization/communication (*M* = 72.80; *SD* = 17.47) and company measures and overall assessment (*M* = 78.61; *SD* = 13.68) achieved mean values of over 70 points, which, in particular, roughly correspond to an affirmation “to a high degree” (Table 4). The dimensions “internal exchange” (*M* = 74.07, *SD* = 18.15) and “working atmosphere” (*M* = 75.46, *SD* = 23.54) achieved the highest mean values in the company organization/communication scale (Table 4). The highest mean values were achieved in the dimensions “workplace as a risk” (*M* = 80.09, *SD* = 23.98) and “crisis-related stress” (*M* = 91.20, *SD* = 24.36) of the “company measures and overall assessment” scale (Table 4). These two dimensions indicate that during the COVID-19 pandemic, heads perceived a high level of workplace risk and crisis-related stress in their workplace (Table 4).

The scale “operational organization and communication” and the psychosocial workload (ER-ratio at work) were moderately negatively correlated, *r* = −0.342, *p* = 0.011 (see Additional File S2). The negative correlation indicates that the ER-ratio decreases as the scale “operational organization and communication” increases. There is also a weak negative correlation between the scale “operational measures and overall assessment” and the ER-ratio, r = −0.285, *p* = 0.037. This negative correlation also indicates that when the scale “operational measures and overall assessment” increases, the ER-ratio of the heads decreases.

The extent to which the subgroups of the study differ from one another is shown in Table 5. There are no significant differences regarding gender (male vs. female) for either scale (*t*(51) = −1.41; *p* = 0.163; *t*(51) = −1.41; *p* = 0.951). Heads “operational organization and communication” did not differ across age groups, *F*(4, 49) = 0.618, *p* = 0.652. The “operational measures and overall assessment” differed significantly between the age groups *F*(4, 49) = 2.926, *p* = 0.030. The 55+ age group had the highest mean values, and the age groups up to 24 years and 35 to 44 years had the lowest mean values (Table 5). There was a low correlation between the company organization/communication scale and the head’s resilience (RS-13), *r* = 0.298, *p* = 0.029 (see Additional File S2). There was a strong correlation between the company organization and communication scale and the operational measures and overall assessment scale, *r* = 0.507, *p* < 0.001 (see Additional File S2).

### 3.6. Pandemic Plans and Involvement in Taskforces

When asked whether the municipal public health authorities had an up-to-date pandemic plan to guide the work of the municipal taskforces, 67.3% (*n* = 35) of the heads answered no. A current pandemic plan was available in 32.7% (*n* = 17). A total of 80% of heads from Bavaria reported that no pandemic plan against a pandemic was in place, followed by heads from Thuringia with 77.7% and those from North Rhine-Westphalia with 57.8%. The correlation between the current pandemic plan and the regional origin of department heads was statistically non-significant (χ^2^ = 0.233; *p* = 0.562).

A total of 98.2% (*n* = 54) of the heads reported that they were actively involved in the decisions of the municipal taskforce as representatives of the municipal public health authority, and 1.8% (*n* = 1) were not involved. In addition, 73.2% of the heads reported that the work of the taskforce had not been evaluated, and 26.8% reported that an evaluation had taken place. Also, 75.9% of the heads did not receive regular crisis management training before the pandemic. In contrast, 24.1% of the heads had received crisis management team training.

In a point-biserial correlation, it was calculated that the active involvement of the heads in the municipal taskforces and the psychosocial workload (ER-ratio at work) were positively correlated. Increased involvement in decision-making in crisis teams is associated with an increase in the ER-ratio. According to Cohen (1992) [17], the variable ER-ratio and the variable involvement in crisis teams show a strong positive correlation, with *r* (53) = 0.658; *p* < 0.0001. 

### 3.7. Deployment of Outreach Teams in Future Crises

The use of outreach teams could be a possible strategy to positively influence the ER-ratio of heads. Therefore, the heads were also asked how important it would be for them to be able to request outreach services in future crises, for example, interdisciplinary and interprofessional teams, so-called outreach teams, which should support citizens, but also medical and care institutions and schools in future crises. A total of 42.3% of the heads considered the use of outreach teams in future crises to be very important, 44.2% somewhat important, 7.7% not very important, 3.8% not at all important, and 2% could not judge. 

**Figure 3:**
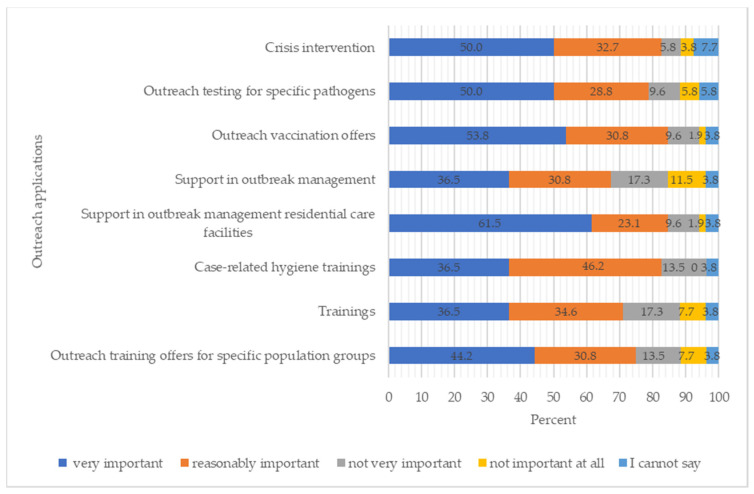
Future deployment options for outreach teams to relieve the burden on municipal public health authorities (*n* = 52).

The survey also asked where the heads saw future needs for the use of outreach teams to support the municipal public health authorities. When asked in which areas the outreach teams should be deployed, crisis intervention (50.0%), testing for specific pathogens (50.0%), vaccination services (53.8%), and support for outbreak management in inpatient care facilities (61.5%) were identified as very important. (Figure 3).

## 4. Discussion

This study provides new information on the psychosocial workload and resilience of heads of municipal public health authorities at the German federal state level during the COVID-19 pandemic. The results show a general trend towards psychosocial stress and strain in the professional group studied. The results of the study show that the surveyed heads experienced a high level of psychosocial workload, had high personal resilience resources, and that the operational organization and communication, as well as the operational measures and overall assessment, were highly effective. The study by Limbrecht-Ecklundt et al. (2015) found a significantly lower ER-ratio for doctors (*M* = 0.71, *SD* = 0.41) [13]. Only 11.9% of the doctors surveyed had an ER-ratio of ≥1 [13]. In the present study, 96.7% of the heads had an ER-ratio > 1. It can be assumed that the majority of heads perceived an imbalance between effort and reward during the COVID-19 pandemic. Shah et al. (2020) also reported that past epidemics such as SARS, MERS, Influenza, and Ebola have led to an increase in mental illnesses such as post-traumatic stress disorder, depression, and anxiety disorders among medical staff [35].

Based on the moderate to high resilience of the heads, it can be assumed that resilient behaviours had a positive impact on the perceived workload during the COVID-19 pandemic. It is important to note that resilience should be considered in a multifactorial way. This means that resilient behaviour should not only be considered individually, but also at the level of groups or organizations. It can also be assumed that general life satisfaction had a positive influence on the resilience of the heads. An increased tendency to spend led to lower life satisfaction among the heads.

The study shows that, overall, female heads reported more work-related stress (ER- ratio at work) and less resilience than their male counterparts. However, there were no significant differences between the sexes for work-related stress (ER-ratio at work) and resilience characteristics (RS-13). Several studies have also identified female gender as a stress and risk factor during epidemics [7,35,36]. Nurminen et al. (2008) already found that there is a high level of job insecurity, especially among female workers [37]. With regard to the qualifications of the heads, it was also found that the mean values of stress resilience (RS-13) and ER-ratio were almost identical. At the German federal state level, the heads from Thuringia, Bavaria, and North Rhine-Westphalia perceived the highest workloads. The highest RS-13 mean values were reported by heads from Berlin and North Rhine-Westphalia. It should also be noted that a higher level of resilience and a lower perceived workload are found among heads responsible for larger districts/counties. This may indicate that municipal public health authorities responsible for larger districts/counties also have a more resilient overall organization. 

It is shown that the high perceived psychosocial workload of the heads is related to the challenging working conditions in the health authorities, e.g., lack of human resources to deal with the pandemic, bureaucracy, and administrative tasks, lack of resources such as budget and equipment. As heads are seen as an important potential source of resources, they should be informed and supported. They should also be trained in appreciative esteem and supportive communication. The role of managers is also highlighted in the WHO’s recommendations for reducing mental stress in healthcare workers during the COVID-19 pandemic [38].

As mentioned above, this study shows that larger districts/counties also have a more resilient overall organization. The next step would be to identify the cause of this structural advantage and whether/how it can be applied to smaller districts. 

After-action reviews (AAR) could be used after a crisis event to develop optimized risk and crisis management, thereby reducing the workload and optimizing the resilience of individuals and institutions. AARs are qualitative, structured reviews of actions taken in response to a specific crisis event [39]. The aim of the review is to identify immediate and longer-term corrective actions for future responses [39]. AARs serve as a tool for identifying and documenting best practices and challenges in a crisis management process [39,40]. The AAR is conducted in four steps: First, the originally planned target state, i.e., the learning objectives of a teaching-learning process or a project, is determined together with the learners. Then, the process is worked through chronologically. Learners reflect not only on observable actions, but also on their mood, expectations, and feelings [41]. The third step involves a target/actual comparison. The group identifies the causes of successes or failures in achieving the goal [41]. The result of this analysis is a summary of the “lessons learned”—in the fourth step [41]. The added value of an AAR therefore lies in the emphasis on collective learning and the development of recommendations for action [41,42]. Moderation plays a central role in the implementation of an AAR. The WHO and also the ECDC provide thematically sorted sets of guiding questions and moderation instructions for this purpose [38,42,43]. 

However, further research is needed to better understand the context, including the needs of the employees of municipal public health authorities, and to develop optimized pandemic management for the municipal public health authorities. Outreach teams operating at the municipal or supra-regional level could provide possible support. These needs in this regard were confirmed by the heads surveyed. However, these needs vary, e.g., in terms of the number of inhabitants for whom the respective municipal public health authorities are responsible and other regional circumstances. In a pandemic, resources are lacking everywhere at the same time. It is therefore important to plan for scalable systems in order to quickly recruit, train, and familiarise the required personnel and set up an outreach team. One possibility would be for smaller health authorities to collaborate or merge during a pandemic or crisis. 

In addition, there is a lack of quantitative and qualitative findings from studies conducted during or after epidemics and pandemics that can serve as a basis for the selection of measures that have a positive impact on the resilience and mental health of frontline employees [1]. For example, Pollock et al. (2020) found no robust evidence on the effectiveness of different strategies to support the resilience of healthcare workers [1]. They only found a few indications of factors that could contribute to the successful implementation of interventions. The review and the present study highlight the need for further studies to develop preventive strategies to maintain the mental health of heads in future pandemic and crisis situations. It is also necessary to prepare such studies in inter-pandemic phases so that they can be more easily carried out in the next pandemic. The long-term course of psychosocial effects after epidemics requires long-term planned support services [36]. It should be critically noted that the time between the online survey and the coronavirus pandemic was quite long. This may have biased the heads’ perceptions. The survey only included heads who were in a managerial position during the coronavirus pandemic and are still in that position. Other professionals working in a public health authority during the pandemic were not surveyed. No distinction was made between the level of support between employees and heads. It is not possible to say whether the heads were perceived as less supportive/overburdened. However, this should be addressed in the IAR in order to find out where the problems to be solved/the potential for improvement lies. Due to the low representativeness of the sample in certain federal states, where heads participated in the survey, the generalizability of the results is limited. The low participation in some of the federal states can be attributed to four factors: The frequent surveys on pandemic topics during the investigation period likely led to survey fatigue. Additionally, the current study addressed pandemic topics that some of the heads may have been reluctant to comment on. The low number of heads in some of the surveyed federal states can also be explained by the limited number of employed heads in those areas. Furthermore, the questionnaire was very extensive, which could have led to survey dropouts.

## 5. Conclusions

Identifying the profile of the heads helps to identify the type of doctors who worked as heads in German municipal public health authorities during the COVID-19 pandemic. In addition, the present study was able to demonstrate that the heads perceived a high psychosocial workload and had high personal resilience resources. The perceived workload is outside the norm for the majority of heads (ER-ratio > 1). It can be assumed that the heads of municipal public health authorities in larger cities, urban districts, or rural districts also work in more resilient organizational structures, which may indicate organizational resilience. In addition, the ER-ratio was lower for heads in larger cities, counties, or districts than for heads in smaller cities, counties, or districts.

However, further research is required to better understand the context, including the needs of the heads of municipal public health authorities, in order to develop an optimized pandemic and crisis management for municipal public health authorities in Germany and to better respond to the perceived workload of the German public health service. In the event of future pandemics and crises, staffing levels in municipal public health departments should be scalable, as they will need to be higher than in normal circumstances.

## Figures and Tables

**Table 1:** Descriptive statistics (*n* = 74).

Characteristics	*n*	%
Gender		
Female	40	54.1
Male	33	44.6
Diverse	1	1.3
Age		
Up to 24 years	2	2.7
25–34 years	4	5.4
35–44 years	10	13.5
45–54 years	17	23.0
55 years and older	41	55.4
Federal states		
Bavaria	18	24.3
North Rhine-Westphalia	32	43.3
Berlin	6	8.1
Saxony	6	8.1
Thuringia	12	16.2

**Table 2:** Individual items of the RS-13 (*n* = 56).

Items	Response to Self-Assessment	*M*	*SD*	Selectivity
1	If I have plans, I follow them through.	5.48	1.47	0.90
2	I usually manage everything somehow.	5.52	1.52	0.90
3	I don’t let myself get thrown off track so easily.	5.84	1.21	0.89
4	I like myself.	5.61	1.31	0.90
5	I can manage several things at the same time.	5.63	1.36	0.90
6	I am determined.	6.04	1.22	0.89
7	I take things as they come.	5.34	1.63	0.90
8	I retain an interest in many things.	5.27	1.57	0.89
9	I can usually look at a situation from several perspectives.	5.95	1.19	0.90
10	I can also overcome myself to do things that I don’t really want to do.	5.34	1.41	0.90
11	When I’m in a difficult situation, I usually find a way out.	5.93	1.05	0.89
12	I have enough energy to do everything I need to do.	5.30	1.48	0.89
13	I can accept it if not everyone likes me.	5.43	1.46	0.90

Note: Likert scale level 1 (disagree) to level 7 (strongly agree).

**Table 3:** Sum and mean values of psychosocial workload according to ERI at work in relation to gender (*n* = 62).

Scales ERI at Work Incl. Overcommitment at Work, ER-Ratio at Work	Women		Men		Miscellaneous		Total	
	(*n* = 31)		(*n* = 30)		(*n* = 1)		(*n* = 62)	
	*M*	*SD*	*M*	*SD*	*M*	*SD*	*M*	*SD*
Effort at work	11.32	1.66	11.56	0.67	12.00	N/A	11.45	1.26
(Range 3 to 12)								
Reward at work	17.68	4.18	19.07	3.73	17.00	N/A	18.34	3.96
(Range 7 to 28)								
Reward at work subscale	5.00	1.75	5.70	1.60	4.00	N/A	5.32	1.69
Esteem								
(Range 2 to 8)								
Reward at work subscale	7.10	2.13	7.43	2.23	8.00	N/A	7.27	2.15
Job promotion								
(Range 3 to 12)								
Reward at work subscale	5.58	1.23	5.93	1.46	5.00	N/A	5.74	1.34
Job security								
(Range 2 to 8)								
Overcommitment at work	19.48	3.54	19.13	3.68	14.00	N/A	19.23	3.62
(Range 6 to 24)								
ER-ratio at work	1.61	0.63	1.47	0.31	1.64	N/A	1.54	0.50

**Table 4:** Mean values of the COVID-19 add-on module for the COPSOQ (*n* = 54).

Scale/Dimension	Item	*M*	*SD*
**Scale: Organization/Communication**		72.80	17.47
Internal exchange	The exchange/communication with my colleagues and managers worked well at the time.	74.07	18.15
Support during the crisis	My colleagues and managers gave me the support I needed to overcome the challenges I faced at the time.	73.61	22.99
Emotional support	I felt that the emotional support I received from my colleagues and managers was sufficient at the time.	68.06	24.96
Working atmosphere	Despite the coronavirus crisis, there was a good working atmosphere in my team/my department at the time.	75.46	23.54
**Scale: operational measures and overall assessment**		78.61	13.68
Internal crisis communication	I felt well informed by my location about the planned and implemented operational measures regarding the corona crisis.	68.98	21.68
Sense of security through measures	Thanks to the protective measures taken at our site with regard to the SARS-CoV-2 virus, I felt well-protected at my workplace.	76.85	18.08
Individual crisis awareness	I thought the hygiene measures implemented or planned at our site to contain the pandemic were excessive.	75.93	27.88
Workplace as a risk	I was worried that I would bring the SARS-CoV-2 virus home from work and put myself and my private environment (family, friends) at risk.	80.09	23.98
Crisis-related stress	I am currently much more worried about my job than I was in the months before the COVID-19 pandemic.	91.20	24.36

Note: 5-point Likert scale from level 0 (to a very low degree) to level 100 (to a very high degree).

**Table 5:** Mean values of the COVID-19 additional module by subgroup for the COPSOQ.

Feature	Group	Organization/Communication *M* (*SD*)	Operational Measures and Overall Assessment *M* (*SD*)
Gender *n* = 74	Female	69.67 (16.32)	78.88 (15.27)
	Male	76.44 (18.48)	78.65 (12.29)
	Diverse	62.50	70.00
Age group *n* = 74	Up to 24	50.00	50.00
	25–34	75.00 (25.00)	76.66 (5.77)
	35–44	68.75 (23.79)	70.00 (20.15)
	45–54	72.72 (16.36)	78.63 (11.63)
	55 and older	74.58 (15.47)	82.33 (10.72)
Qualification *n* = 64	Medical specialist qualification for public health care	71.02 (15.84)	80.60 (12.29)
	Other medical specialist qualification	72.85 (18.41)	81.09 (13.36)
	Degree in public health	68.75 (17.03)	79.99 (12.53)

Note: 5-point Likert scale from level 0 (to a very low degree) to level 100 (to a very high degree).

## Data Availability

The data sets used and/or analyzed during the current study are available from the corresponding author on reasonable request.

## Supplementary Materials

The following supporting information can be downloaded at: https://www.mdpi.com/article/10.3390/ijerph21111421/s1, Additional File S1: Graphical abstract heads of municipal public health authorities; Additional File S2: Table S1. Correlation coefficients.

## Abbreviations

AAR	After Action Review
COPSOQ	Copenhagen Psychosocial Questionnaire
ECDC	European Center for Disease Prevention and Control
ERI	Effort-Reward Imbalance
IBM	International Business Machines Corporation
MERS	Middle East Respiratory Syndrome
RS-13	Resilience Scale-13
SARS	Severe Acute Respiratory Syndrome
SPSS	Statistical Package for the Social Sciences
L-1	General Life Satisfaction Short Scale
WHO	World Health Organization
