# Supplementary material for: Psychosocial Workloads and Resilience of Heads of Municipal Public Health Authorities in Germany During the COVID-19 Pandemic: Perceptions of Operational Organization, Communication, and Measures
Source: Int J Environ Res Public Health. 2024 Oct 26;21(11):1421. doi: 10.3390/ijerph21111421 (PMC11594032; doi:10.3390/ijerph21111421)
Supplement: Supplementary file 1 [file ijerph-21-01421-s001.zip › Additional_file_s2_Table_Correlation_coefficients.pdf]

Table S1. Correlation coefficients among the study variables

Variables	(1)	(2)	(3)	(4)	(5)
(1) ER-ratio	1				
(2) RS-13	-.151	1			
(3) Overcommitment	.485***	.024	1		
(4) Organization/communication	-.342*	.298*	-.114	1	
(5) Operational measures and overall assessment	-.285*	.229	.029	.507***	1

Note: ER-ratio (Effort/Reward ratio), * $p < 0.05$, ** $p < 0.01$, *** $p < 0.001$
