# Supplementary figures and images for: Psychosocial Workloads and Resilience of Heads of Municipal Public Health Authorities in Germany During the COVID-19 Pandemic: Perceptions of Operational Organization, Communication, and Measures
Source: Int J Environ Res Public Health. 2024 Oct 26;21(11):1421. doi: 10.3390/ijerph21111421 (PMC11594032; doi:10.3390/ijerph21111421)

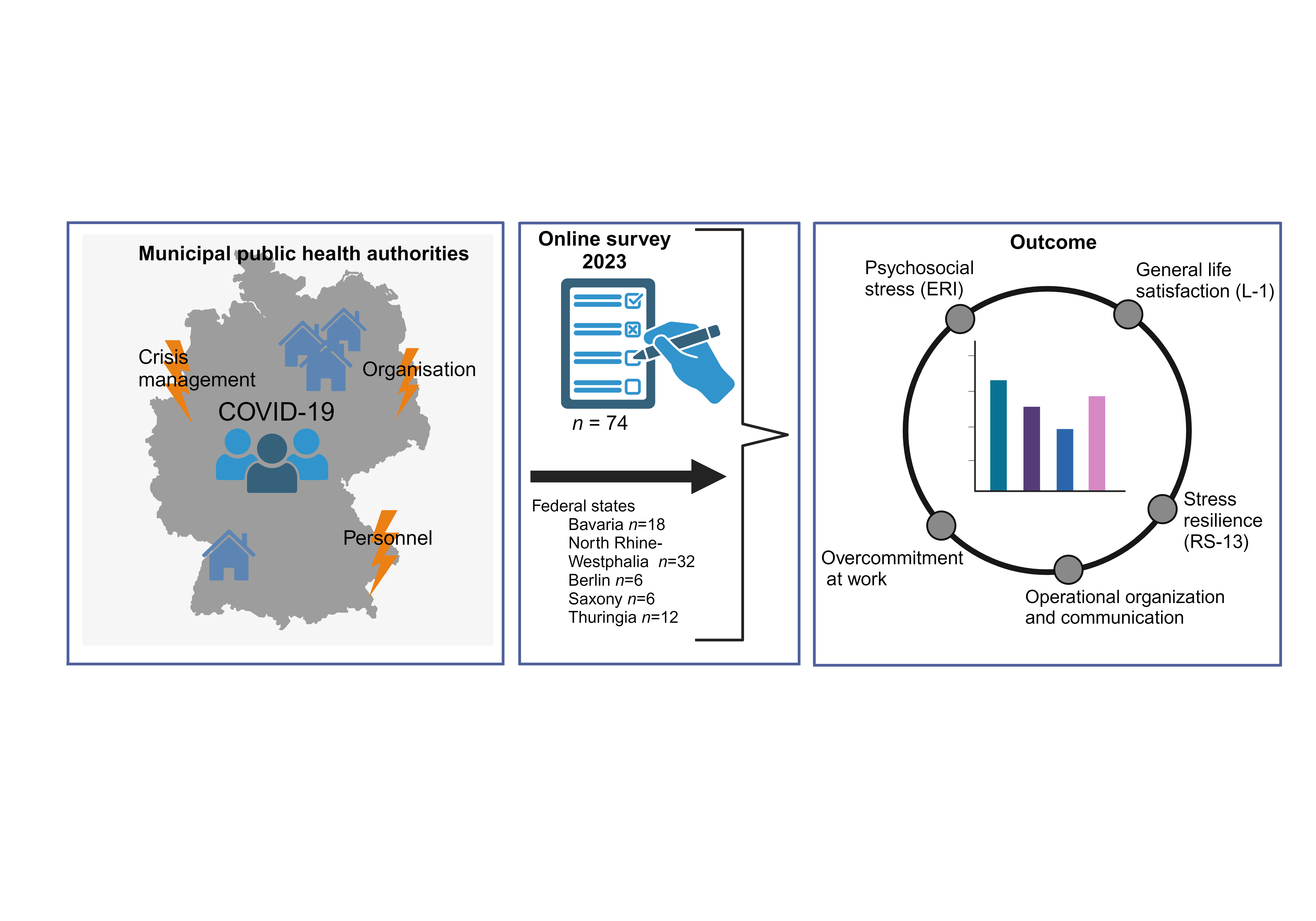

Supplement: Supplementary file 1 [file ijerph-21-01421-s001.zip › Additional_file_s1_graphical_abstract_heads_of_municipal_public_health_authorities.png]
